# A panel of DNA methylation signature from peripheral blood may predict colorectal cancer susceptibility

**DOI:** 10.1186/s12885-020-07194-5

**Published:** 2020-07-25

**Authors:** Justina Ucheojor Onwuka, Dapeng Li, Yupeng Liu, Hao Huang, Jing Xu, Ying Liu, Yuanyuan Zhang, Yashuang Zhao

**Affiliations:** Department of Epidemiology, Public Health College, Harbin Medical University, 157 Baojian Street, Nangang District, Harbin, 150081 Heilongjiang Province People’s Republic of China

**Keywords:** Colorectal cancer, DNA methylation, Methylation risk score, Peripheral blood

## Abstract

**Background:**

Differential DNA methylation panel derived from peripheral blood could serve as biomarkers of CRC susceptibility. However, most of the previous studies utilized post-diagnostic blood DNA which may be markers of disease rather than susceptibility. In addition, only a few studies have evaluated the predictive potential of differential DNA methylation in CRC in a prospective cohort and on a genome-wide basis. The aim of this study was to identify a potential panel of DNA methylation biomarkers in peripheral blood that is associated with CRC risk and therefore serve as epigenetic biomarkers of disease susceptibility.

**Methods:**

DNA methylation profile of a nested case-control study with 166 CRC and 424 healthy normal subjects were obtained from the Gene Expression Omnibus (GEO) database. The differentially methylated markers were identified by moderated t-statistics. The DNA methylation panel was constructed by stepwise logistic regression and the least absolute shrinkage and selection operator in the training dataset. A methylation risk score (MRS) model was constructed and the association between MRS and CRC risk assessed.

**Results:**

We identified 48 differentially methylated CpGs sites, of which 33 were hypomethylated. Of these, sixteen-CpG based MRS that was associated with CRC risk (OR = 2.68, 95% CI: 2.13, 3.38, *P* <  0.0001) was constructed. This association is confirmed in the testing dataset (OR = 2.02, 95% CI: 1.48, 2.74, *P* <  0.0001) and persisted in both males and females, younger and older subjects, short and long time-to-diagnosis. The MRS also predicted CRC with AUC 0.82 (95% CI: 0.76, 0.88), indicating high accuracy.

**Conclusions:**

Our study has identified a novel DNA methylation panel that is associated with CRC and could, if validated be useful for the prediction of CRC risk in the future.

## Background

Colorectal cancer (CRC) poses a great public health concern globally. It is the third most common cancer diagnosed among men and the second most common among women and was responsible for an estimated 1.8 million new cases and 881,000 deaths in 2018 [1]. In the United States of America, CRC is the third most common cancer diagnosed with about 140,250 new cases and 50,630 deaths in 2017 [2]. In addition to environmental factors, there is proven evidence that CRC results from the accumulation of genetic and epigenetic changes, which changes colonic epithelial cells into adenocarcinoma cells [3].

Epigenetic alterations such as DNA methylation has been associated with many human diseases including cancer and have also been reported to occur early in the development of colorectal tumors [3] by playing a role in gene expression and genomic stability. DNA methylation markers show great potential in the detection and diagnosis of cancer [4] and a panel of differential DNA methylation could be a possible biomarker of CRC susceptibility.

Peripheral blood is an easily accessible source of genomic DNA that can be used to estimate DNA methylation profiles and could serve as useful non-invasive and informative biomarkers for cancer risk [5]. Several studies have investigated peripheral blood DNA methylation biomarkers in different cancer types including head and neck, urothelial, breast, lung, bladder, gastric cancer, prostate, and ovarian cancers [6–16]. Some epidemiologic studies have assessed peripheral blood DNA methylation biomarkers in CRC. However, most of the studies used post-diagnostic blood DNA which may imply that DNA methylation alterations could be an early response of the hematologic system to the presence of CRC cells [17, 18]. The few studies that utilized pre-diagnostic DNA focused on genomic methylation of leukocyte DNA [19, 20] while other studies involved candidate genes [21–23] and methylation at repetitive elements [24]. There are few genome-wide DNA methylation studies that have evaluated the association of pre-diagnostic peripheral blood DNA with CRC risk.

In order to identify a potential panel of DNA methylation biomarkers in peripheral blood that are associated with CRC risk and therefore serve as epigenetic biomarkers of disease susceptibility, we performed an epigenome-wide analysis of a nested case-control study using peripheral blood Illumina HumanMethylation450 bead-array DNA methylation data. We repurposed data previously analysed by Cordero et al who focused on probes associated with genes encoding for miRNAs [25]. We analysed the data using two methods including epigenome-wide methylation profiling to identify differentially methylated CpGs as well as a machine learning algorithm to construct a sixteen-CpG based methylation risk score predictive of CRC risk.

## Methods

### Data source

The Illumina Human Methylation 450 Beadchip data of the Italian arm of the European Prospective Investigation into Cancer and Nutrition (EPIC-Italy) were obtained from Gene Expression Omnibus (GEO) with the accession number GSE51032. The EPIC is a multicenter prospective study aimed at investigating the complex relationships between nutrition and various lifestyle factors and the etiology of cancer and other chronic diseases [26]. The EPIC-Italy cohort that was produced in Turin, Italy, is a sub-cohort that comprised of 46,857 volunteers, recruited from five different centers within Italy (Varese, Turin, Florence, Naples and Ragusa) with standardized lifestyle and personal history questionnaires, anthropometric data as well as blood samples collected for DNA extraction. At the last follow-up (2010), 424 participants remained cancer-free, 166 had developed primary colorectal cancer. We extracted the data containing the DNA methylation status of 485,512 CpG sites in the 166 participants who had developed primary colorectal cancer and the 424 matched cancer-free participants.

### DNA methylation profiling in CRC and healthy normal subjects

The differential methylation analysis was conducted using the workflow by Maksimovic et al. [27]. Briefly, we pre-processed and normalized the data using R package minfi [28]. The quality control, pre-filtering were conducted with the minfi package and the Functional Normalization (FunNorm) function was used for normalization [28, 29]. Quality control was performed and probes with detection *P*-value > 0.01 in at least one sample were filtered out. After normalization, all probes containing single nucleotide polymorphism (SNPs) and probes mapped to sex chromosomes were filtered out to prevent bias due to unknown genetic background and mixed gender of samples, respectively. Cross-reactive probes, which refer to probes that have shown to map to several positions in the genome [30] were also filtered out. After normalization and quality control, the probes yielded were used for further analysis.

### Hierarchical clustering

We conducted Hierarchical clustering using complete linkage with a Euclidian distance in the R package pheatmap [31].

### Functional analysis

In order to examine main biological functions that were controlled by DNA methylation, we used DMPs (differentially methylated positions) for Gene ontology (GO) analyses and Kyoto Encyclopedia of Genes and Genomes (KEGG) based on the gometh function in the R package missMethyl [32].

### Selection of differentially methylated markers for risk model

The methylation level of all the probes was indicated as beta (β) values, which is the proportion of the methylated probe intensity to the total probe intensity (sum of methylated and unmethylated probe intensities plus constant α, where α = 100). The beta values for CRC and healthy normal subjects were log-transformed to obtain the M-values and used for further analysis, with the beta values used for visualization while the M-values were used for statistical analysis which is in conformity with Du et al. [33]. The linear models for microarray data (LIMMA) package was used to identify differentially methylated genes between CRC cases and healthy normal subjects [34]. Moderated t-test and mean methylation value differences (delta (∆) beta) were generated and we corrected *P* values of individual probe for multiple testing using the Benjamini-Hochberg method. A CpG site between CRC and healthy normal subjects was considered significant with a false discovery rate (FDR) <  0.05 and ∆β ≥ 5% and DMPs.

In addition, DMPs were used to build a risk score model. The entire sample of 590 was randomly split into 70% training and 30% testing sets using stratified random sampling by case-control status. The stratification was to guarantee an equal distribution of CRC and healthy normal subjects between sets, prevent overfitting the data, and allow for validation of the model. The stepwise logistic regression and least absolute shrinkage and selection operator (LASSO) [35] methods were then applied on the training set to select the best markers for CRC prediction using R packages MASS and glmnet respectively [36, 37]. For the LASSO selection analysis, we used 10-fold cross-validation to identify the tuning parameter and chose the minimum lambda, which is the value of lambda with the smallest mean cross-validated error. Nineteen CpGs were identified by using the stepwise regression method and twenty-two CpGs were identified by using the LASSO analysis. In these two approaches, sixteen overlapping markers were identified between the two methods.

### Construction of methylation risk score

Logistics regression models were fitted on the training dataset using these sixteen markers and MRS for each patient was calculated. The calculation was carried out by multiplying the methylation level for each CpG site with the corresponding regression coefficient and summed over all CpG sites as follows:

$$ \mathrm{MRS}={\upbeta}_1{\mathrm{x}}_1+{\upbeta}_2{\mathrm{x}}_2+{\upbeta}_3{\mathrm{x}}_3+\dots \dots \dots \dots .+{\upbeta}_{\mathrm{k}}{\mathrm{x}}_{\mathrm{k}} $$

Where *β* represents the estimated regression coefficient of the CpG site k derived from the logistic regression analysis, and *x* represents the methylation level of the CpG site k.

Furthermore, we determined whether our findings could be validated in the testing dataset. The MRS was constructed on the training set and validated on the testing set by fitting a logistic regression model to determine the association of the MRS with CRC, with the MRS added into the model as a continuous variable.

### Subgroup analyses

To assess the robustness of our findings, we determined whether the association between MRS and CRC risk differed by gender, age, and time-to-diagnosis by conducting subgroup analyses according to these variables both in the training and testing datasets. We took advantage of the prospective design of this study and explored the effect of time-to-diagnosis. We categorized the CRC subjects into short (less than 6 years) and long (above 6 years) time-to-diagnosis using the median as a cut-off. In addition, we conducted a case-only analysis and assessed whether methylation levels of the CpGs were correlated with time-to-diagnosis (the time interval between blood draw and diagnosis of CRC).

### External validation in TCGA tissues

In order to validate the predictive performance of the sixteen-CpG panel MRS in an independent dataset, we analysed the CRC data in TCGA (The Cancer Genome Atlas) dataset. The level 3 DNA methylation data detected by HumanMethylation450 in colon cancer and rectal cancer were downloaded from UCSC Xena (https://xena.ucsc.edu/). We constructed a univariate logistic regression model using the 13-CpGs differentially methylated in TCGA.

### Statistical analysis

The distribution of the demographic characteristics in the study group was compared between CRC and healthy normal subjects using Chi-square and Kruskal–Wallis tests for categorical and continuous data respectively. To estimate the difference in methylation level between CRC and healthy normal, two-sample t-tests (moderated t-tests) with Bonferroni correction was performed for each CpG. Univariate and multivariate logistic regression were used to estimate odds ratios (ORs) and corresponding 95% confidence intervals (CI) for DNA methylation and MRS between CRC and healthy normal subjects, as well as subgroup analysis. The ROC curves were plotted with R package pROC version 1.16.1 [38], to estimate the discriminatory power of the MRS. The area under the ROC curve (AUC) was calculated and the DeLong method was used to calculate the 95% confident interval (CI) for AUC. The Correlation was performed using Pearson’s method. The significance level used for all tests was two-tailed *P* <  0.05. All statistical analyses were carried out using R language software version 3.5.1 (https://cran.r-project.org/bin/windows/base/old/3.5.1/).

## Results

### Identification of differentially methylated markers

**Fig. 1:**
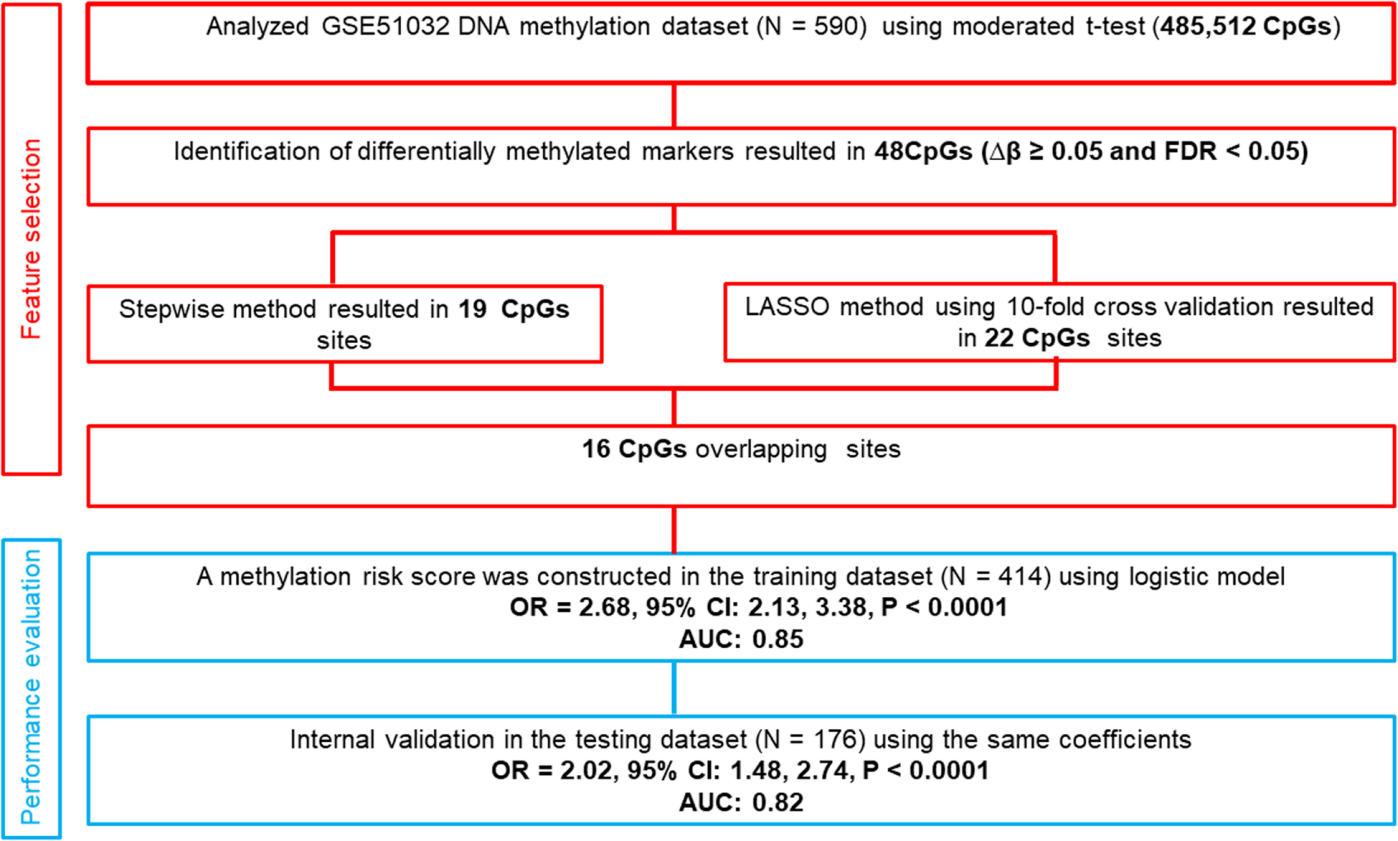
Overall workflow of the step-by-step analyses process of this study

The workflow showing the step-by-step procedure for this analysis and the demographic characteristics of participants are presented in Fig. 1 and Table 1 respectively. We analysed the microarray methylation profile of 166 (87 males and 79 females) CRC and 424 (84 males and 340 females) healthy normal subjects. The average age of CRC subjects was 55 years old whereas, the normal subjects had a mean age of 53. CRC and healthy normal subjects were statistically significantly different with respect to gender but did not differ with respect to age. We adjusted for age and gender in our models. The average time-to-diagnosis for cases was 6.2 years (range = 0–14.3). The Illumina Human Methylation 450 Beadchip contained the DNA methylation status of 485,512 CpG sites. Pre-processing and quality control were performed and the poor performing probes were filtered out. A total of 399,934 CpG sites (Additional file 1: Figure S1) were yielded, and their methylation data were used for further analysis. A total of 49,299 CpGs (corresponding with 11, 786 unique genes) were differentially methylated (*FDR* <  0.05) between the CRC and healthy normal subjects.

**Table 1:** Characteristics of Training and Testing Dataset of Nested Case Control Study Based on EPIC-Italy Cohort

Characteristics	Entire Dataset		Training Dataset		Testing Dataset	
	Cases	Control	Cases	Control	Cases	Control
Total	166	424	117	297	49	127
Age, Mean (SD)	55.07 (6.73)	53.23 (7.19)	55.94 (6.73)	53.08 (7.20)	55.25 (6.62)	53.56 (7.20)
< 60	128 (26.9)	348 (73.1)	89 (26.4)	245 (73.4)	10 (27.5)	103 (72.5)
≥ 60	38 (33.3)	76 (66.7)	28 (35.0)	52 (65.0)	39 (29.4)	24 (70.6)
Gender						
Male	87 (52.4)	84 (19.8)	55 (47.4)	61 (52.6)	32 (58.2)	23 (41.8)
Female	79 (47.6)	340 (80.2)	62 (20.8)	236 (79.2)	17 (14.0)	104 (86.2)
Time-to-diagnosis (years)						
< 6	80 (48.2)	NA	56 (47.9)	NA	24 (49.0)	NA
≥ 6	86 (51.8)	NA	61 (52.1)	NA	25 (51.0)	NA

*Abbreviations*: *NA* Not applicable, *SD* Standard deviation

Gene Ontology (GO) terms and KEGG pathway enrichment analysis for genes associated with the 49,299 differentially methylated CpGs were performed. The GO analysis showed the molecular functions, cellular components, and biological functions of differentially methylated genes under the criterion *FDR* <  0.05 (Additional file 2: Table S1). In the KEGG pathway genes showed enrichments in the metabolic pathway *(FDR* = 1.19e-03), cancer- pathways (*FDR* = 6.58e-03), human papillomavirus infection (*FDR* = 1.61e-02), Rap1 signaling pathway (*FDR* = 4.36e-04) and Axon guidance (*FDR* = 2.12e-03) (Additional file 3: Table S2).

**Fig. 2:**
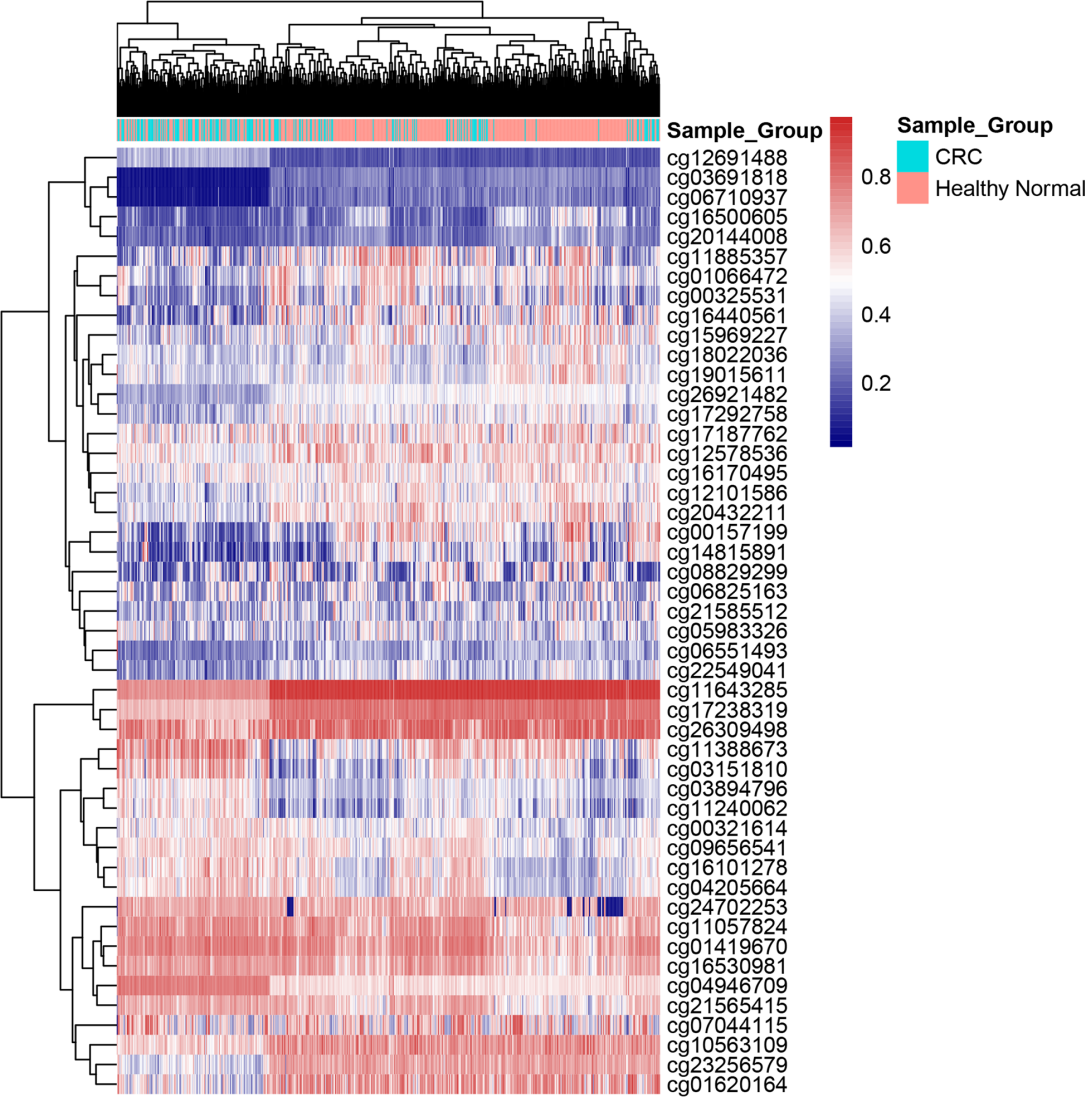
The heatmap showing the methylation levels of 48 CpG sites differentially methylated in the entire dataset. Row represents specific markers (*N* = 48). Column represents samples (*N* = 590)

Of the 49,299 CpGs differentially methylated, 48 CpGs (corresponding with 29 unique genes) which had absolute mean β-value difference (|∆β| ≥ 0.05) were selected and denoted DMPs (Additional file 4: Table S3). Among the DMPs, a total of 15 CpGs (corresponding with 8 unique genes) were hypermethylated and 33 CpGs (corresponding with 21 unique genes) were hypomethylated. Hierarchical clustering was implemented to determine whether the identified DMPs could distinguish CRC from healthy normal subjects. The results showed a significant difference in methylation between CRC and healthy normal subjects (Fig. 2).

### Methylation risk score construction

The entire sample of 590 was randomly split into training (117 CRC subjects and 297 healthy normal subjects) and testing (49 CRC subjects and 127 healthy normal subjects) sets (Table 1). Differentially methylated markers associated with CRC risk were screened on the training dataset using LASSO selection and stepwise logistic regression analysis. The sixteen markers mapped to nine genes including *LGR6, PTPN12, PPFIA3, LOC399959, PCDHGA1, RNF39, ESYT3, MRGPRG, and ATHL1* overlapping between the two methods were selected (Additional file 5: Figure S2). The associations of the sixteen individual markers with CRC by univariate and multivariate logistic regression analysis are presented in Additional file 6: Table S4 and Table 2 respectively.

**Table 2:** Multivariate Analysis on the Associations of DNA Methylation Marker, MRS and Risk of CRC of Nested Case Control Study Based on EPIC-Italy Cohort

CpG ID	Gene Name	Entire Dataset			Training Dataset			Testing Dataset		
		OR	95% CI	*P-value*	OR	95% CI	*P-value*	OR	95% CI	*P-value*
cg06551493	PTPN12	0.62	0.49, 0.78	**6.58e-05**	0.71	0.54, 0.91	**0.009**	0.42	0.25, 0.68	**7.18e-04**
cg01419670	NA	2.12	1.62, 2.85	**1.47e-07**	2.36	1.71, 3.36	**6.16e-07**	1.62	1.00, 2.82	0.06
cg16530981	NA	1.96	1.52, 2.57	**5.01e-07**	2.15	1.60, 2.98	**1.29e-06**	1.53	0.99, 2.57	0.08
cg18022036	NA	0.56	0.45, 0.70	**5.47e-07**	0.54	0.41, 0.69	**3.97e-06**	0.67	0.43, 1.03	0.07
cg12691488	NA	0.73	0.47, 1.10	0.14	0.67	0.40, 1.08	0.11	0.84	0.34, 1.95	0.70
cg17292758	PPFIA3	0.79	0.64, 0.98	**0.04**	0.79	0.61, 1.02	0.07	0.79	0.51, 1.20	0.27
cg16170495	RNF39	0.66	0.54, 0.80	**3.17e-05**	0.68	0.54, 0.85	**0.001**	0.62	0.41, 0.90	**0.01**
cg11240062	NA	1.25	1.00, 1.57	0.06	1.30	1.00, 1.69	0.05	1.11	0.70, 1.78	0.67
cg21585512	LOC399959	0.68	0.55, 0.83	**1.61e-04**	0.58	0.45, 0.74	**1.64e-05**	0.94	0.64, 1.38	0.75
cg24702253	MRGPRG	1.74	1.28, 2.58	**0.002**	1.78	1.24, 2.81	**0.005**	0.74	1.01, 4.58	0.11
cg17187762	NA	0.76	0.63, 0.93	**0.006**	0.78	0.62, 0.97	**0.03**	0.66	0.44, 0.98	**0.04**
cg05983326	PCDHGA1	0.69	0.57, 0.84	**2.68e-04**	0.73	0.57, 0.91	**0.007**	0.57	0.38, 0.84	**0.006**
cg06825163	LGR6	0.70	0.57, 0.86	**5.47e-04**	0.67	0.52, 0.84	**8.11e-04**	0.82	0.56, 1.21	0.34
cg11885357	ESYT3	0.89	0.73, 1.08	0.23	0.83	0.65, 1.04	0.11	1.07	0.72, 1.59	0.74
cg08829299	ATHL1	0.86	0.70, 1.04	0.13	0.84	0.66, 1.05	0.13	0.93	0.63, 1.37	0.69
cg07044115	NA	0.77	0.63, 0.93	**0.008**	0.82	0.66, 1.03	0.07	0.60	0.40, 0.89	**0.01**

*Abbreviations*: *CI* Confidence interval, *CRC* Colorectal cancer, *MRS* Methylation risk score, *ORs* Adjusted for age and gender, *P* values < 0.05 are in bold

Furthermore, using the sixteen-CpG panel we calculated a methylation risk score (MRS) for each subject on the training dataset using the formula:

$$ \mathrm{MRS}=\left(-0.4100\ast \mathrm{cg}06551493\right)+\left(0.4332\ast \mathrm{cg}01419670\right)+\left(0.2895\ast \mathrm{cg}16530981\right)+\left(-0.5172\ast \mathrm{cg}18022036\right)+\left(-0.3915\ast \mathrm{cg}12691488\right)+\left(-0.3246\ast \mathrm{cg}17292758\right)+\left(-0.2886\ast \mathrm{cg}16170495\right)+\left(0.2451\ast \mathrm{cg}11240062\right)+\left(-0.5651\ast \mathrm{cg}21585512\right)+\left(0.3615\ast \mathrm{cg}24702253\right)+\left(-0.2445\ast \mathrm{cg}17187762\right)+\left(-0.3951\ast \mathrm{cg}05983326\right)+\left(-0.5089\ast \mathrm{cg}06825163\right)+\left(-0.2504\ast \mathrm{cg}11885357\right)+\left(-0.2357\ast \mathrm{cg}08829299\right)+\left(-0.3607\ast \mathrm{cg}07044115\right). $$

The methylation levels of 4 CpG (cg01419670, cg16530981, cg11240062, cg24702253) sites were hypermethylated, and 12 CpG (cg06551493, cg18022036, cg12691488, cg17292758, cg16170495, cg21585512, cg17187762, cg05983326, cg06825163, cg11885357, cg08829299, cg07044115) sites were hypomethylated.

**Fig. 3:**
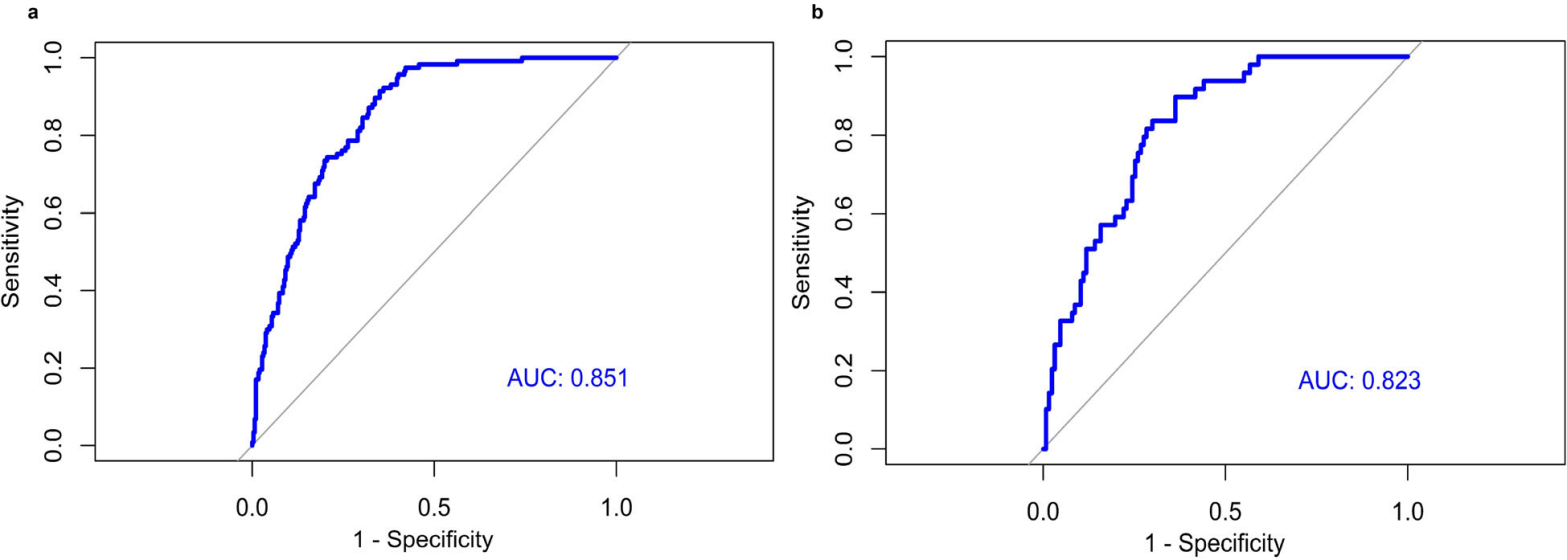
Classification performance of methylation risk score (MRS) for CRC risk (**a**) Receiver operating characteristic (ROC) curve for methylation risk score (MRS) prediction of CRC, with area-under the curve (AUC) of 0.85 (95% CI: 0.82–0.88) on training dataset. **b** Receiver operating characteristic (ROC) curve for methylation risk score (MRS) prediction of CRC, with area-under the curve (AUC) 0.82 (95% CI: 0.76–0.88) on testing dataset

The MRS (range, − 5.59 to 4.35) was significantly higher for CRC subjects than in healthy normal subjects (*P* <  0.000), with a median MRS of 1.68 (IQR, 1.43) in CRC subjects and − 0.430 (IQR, 2.89) in healthy normal subjects (Additional file 7: Figure S3a) in the training dataset. The MRS was associated with a 2.68-fold increased risk of CRC (OR = 2.68, 95% CI: 2.13, 3.38, *P* <  0.0001) Table 2. The MRS showed a good predictive ability for discriminating between CRC and healthy normal subjects (AUC, 0.85; 95% CI: 0.81, 0.89) Fig. 3a.

### Validation of the sixteen-CpG panel MRS for CRC prediction in the testing dataset

In order to validate the predictive performance of the sixteen-CpG panel MRS for the prediction of CRC risk, the predictive model was applied to the testing dataset. The MRS (range, − 5.73 to 3.89) was also significantly higher for CRC subjects than in healthy normal subjects (*P* <  0.0001), with median MRS of 1.83 (IQR, 1.80) in CRC subjects and − 0.45 (IQR, 2.64) in healthy normal subjects (Additional file 7: Figure S3b). Consistent with the training dataset, the MRS was associated with a 2.02-fold increased risk of CRC (OR = 2.02, 95% CI: 1.48, 2.74, *P* <  0.0001) Table 2. Similar to the training dataset, the MRS showed a good predictive ability for discriminating between CRC and healthy normal subjects (AUC, 0.82; 95% C: 0.76, 0.88) Fig. 3b.

### Subgroup analysis for the association between MRS and CRC risk

When the study subjects were stratified according to gender, age and time-to-diagnosis, the MRS still demonstrated an increased risk of CRC among both male and female subjects, younger (< 60 years) and the older (≥ 60 years) subjects as well as short and long time to diagnosis in the training and testing datasets (Table 3). Also, the case-only analysis demonstrated no correlation between methylation levels time-to-diagnosis, with only one CpG showing small negative correlation (Additional file 8: Table S5).

**Table 3:** Associations of MRS and Risk of CRC According to Age, Gender and Time-To-Diagnosis of Nested Case Control Study Based on EPIC-Italy Cohort

Characteristics	Entire Dataset			Training Dataset			Testing Dataset		
	OR	95% CI	*P-value*	OR	95% CI	*P-value*	OR	95% CI	*P-value*
Age									
< 60	2.35	1.95, 2.90	**< 0.0001**	2.62	2.06, 3.44	**< 0.0001**	1.97	1.44, 2.84	**< 0.0001**
≥ 60	2.60	1.77, 4.17	**< 0.0001**	2.87	1.77, 5.35	**0.0002**	2.28	1.20, 5.51	**0.03**
Gender									
Male	1.97	1.47, 2.71	**< 0.0001**	2.10	1.44, 3.21	**0.0002**	1.91	1.20, 3.37	**0.02**
Female	2.64	2.12, 3.37	**< 0.0001**	2.96	2.26, 4.08	**< 0.0001**	2.08	1.46, 3.20	**0.0002**
Time-diagnosis									
< 6 years	2.21	1.80, 2.77	**< 0.0001**	2.40	1.86, 3.20	**< 0.0001**	1.99	1.40, 3.02	**0.0004**
≥ 6 years	2.51	2.01, 3.23	**< 0.0001**	2.88	2.17, 4.01	**< 0.0001**	1.97	1.36, 3.05	**0.0008**

*Abbreviations*: *CI* Confidence interval, *CRC* Colorectal cancer, *MRS* Methylation risk score, *OR* Odds ratios adjusted for age and gender, *P* values < 0.05 are in bold

### Independent validation of the sixteen-CpG panel MRS for CRC prediction in TCGA dataset

We used TCGA dataset of 391 CRC and 45 controls for independent validation of our sixteen-CpG panel MRS. Only thirteen CpGs of the panel were differentially methylated in the TCGA dataset. The beta values of the thirteen CpGs were extracted and univariate logistic regression models were constructed (Additional file 9: Table S6). We identified nine CpGs (cg06551493, cg12691488, cg17292758, cg16170495, cg21585512, cg24702253, cg17187762, cg05983326, cg11885357) that were associated with CRC, and the MRS for each sample was calculated. The MRS (range, − 4.05 to 2.92) was significantly higher for CRC subjects than in controls subjects (*P* < 0.0001), with a median MRS of 0.16 (IQR, 1.59) in CRC subjects and − 0.712 (IQR, 0.95) in controls (Additional file 10: Figure S4). The MRS was associated 1.96-fold increased risk in CRC (OR = 2.06, 95% CI: 1.55, 2.78, *P* < 1.08e-06) (Additional file 9: Table S6). The MRS showed a good predictive ability for discriminating between CRC and control subjects (AUC, 0.73; 95% CI: 0.66–0.79) Additional file 11: Figure S5.

## Discussion

In this study, we repurposed a microarray peripheral blood DNA methylation data of CRC and healthy normal subjects obtained from the GEO database. First, we identified differentially methylated CpGs between CRC and healthy normal subjects for CRC-specific methylation panel. Second, we divided the data into two sets and identified a panel of sixteen CpGs associated with CRC by logistic regression in the training dataset. Third, we constructed a predictive model- MRS, to predict the risk of CRC based on the linear combination of methylation levels of the sixteen CpGs. The MRS was tested first on the training dataset and was associated with the risk of CRC, the prediction evaluation when conducted by ROC analysis attained an AUC of 0.85. Subgroup analyses demonstrated that these significant associations persisted in both males and females, younger and older subjects as well as long and short time-to-diagnosis. The MRS, when validated on the testing dataset attained an AUC of 0.82 indicating that the risk predictive value of the MRS panel is replicable for predicting CRC risk. Our findings show a panel of peripheral blood DNA methylation that is a potential biomarker for CRC susceptibility.

Previous studies have developed multiple gene methylation-based panels to predict an individual’s susceptibility to CRC. For example, Liu et al. and Luo et al. both reported DNA methylation-based panels in blood leukocyte that were associated with 6.51-fold (95% CI, 3.77–11.27) and 1.54-fold (95% CI: 1.15–2.05) increased risk of CRC respectively and this is similar to our result. However, since both studies involved post-diagnostic DNA samples based on case-control studies, the association detected may have resulted from a response to CRC cells rather than CRC susceptibility.

Although the mechanisms underlying the aberrations in the methylation of peripheral blood DNA among individuals who are susceptible to CRC are not clear, our analysis used pre-diagnostic peripheral blood DNA, which indicates that methylation aberrations in peripheral blood DNA could possibly be a long-term CRC predisposition risk markers or a far early response to CRC cells before cancer could be detected by techniques used before now such as endoscopy and cytology. In addition, there was no correlation between DNA methylation and time-to-diagnosis in case-only analysis, which also supports the suggestion that peripheral blood DNA could be a long-term event.

Contrary to our result, the previous studies that utilized pre-diagnostic blood DNA found no association between pre-diagnostic genomic DNA methylation status and CRC risk [19, 20]. This difference might be because of the heterogeneous methodology and assays. The two studies evaluated leukocyte genomic DNA methylation levels by liquid chromatography/tandem mass spectrometry, which only considers DNA hypomethylation and not regional hypermethylation that can also contribute to increased risk of CRC.

The presence of specific single nucleotide polymorphisms (SNPs) has also been used to evaluate an individual’s risk for CRC both by the candidate and multiple genes (by a method called a genetic risk score (GRS)) as well as genome-wide association study (GWAS). Similar to the associations we found between MRS and CRC risk, GRS based on SNPs have been associated with CRC risk. For example, Cho et al. [39], reported a higher GRS that was associated with CRC (OR, 2.57; 95% CI, 1.89, 3.49) using thirteen SNPs. In addition, Jung et al. [40] in a case-cohort study, demonstrated that participants in the highest quartiles of the genetic risk score had an increased risk of CRC (hazard ratio, 2.65; 95% CI, 1.43 to 4.91) compared with those in the lowest quartile using seven SNPs. Furthermore, a GWAS study found a SNPs developed polygenic risk score (PRS) that was associated with about 2-fold increased risk of CRC [41].

In the present study, the methylation-based markers for CRC included *LGR6, PTPN12, PPFIA3, LOC399959, PCDHGA1, RNF39, ESYT3, MRGPRG and ATHL1*, all of which were located in the promoter regions or first introns of nearby genes. There are limited epidemiological reports on the association between these markers and CRC risk. One of the genes, *LGR6* (Leucine-Rich Repeat Containing G Protein-Coupled Receptor 6) regulates the phosphoinositide 3-kinase/AKT signaling pathway and plays a tumor-promoting role in CRC development indicating that it might be a potential diagnostic and prognostic biomarker for CRC [42]. Protein tyrosine phosphatase non-receptor type 12 (*PTPN12*) are signaling molecules that regulate a variety of cellular processes and has been found to be epigenetically regulated in triple-negative breast cancer [43]. They are known to play an important role in cell growth, proliferation, and motility [44] and have been found to function as a suppressor of epithelial cell motility in CRC cells [45]. A study on whole-exome sequencing identified that PTPN12 variant is associated with CRC susceptibility [46]. In addition, the methylation of PTPRF-interacting protein alpha 3(*PPFIA3*) in serum has shown a potential for the detection of gastric cancer [47].

The pathway analysis demonstrated that metabolic pathways, cancer pathways, human papillomavirus infection, Rap1 signaling pathway, and Axon guidance were associated with CRC. The biological processes involve cellular component organization or biogenesis, and cellular localization. The Rap1 signaling pathway has been implicated in the previous genome-wide profile of colorectal cancer [48] and has been known to play several important roles in tumor cell invasion and metastasis [49]. The pathway and biological processes put together demonstrate that multiple pathways, which were affected by aberrant methylation were involved in CRC tumorigenesis.

In order to validate the MRS, we conducted an independent validation analysis of our results using TCGA dataset for CRC risk prediction. Despite the fact that only 9 CpGs from 16-CpG MRS panel was available in TCGA datasets for calculation of MRS, the MRS was still higher for CRC subjects then controls. It is noteworthy that *PTPN12, RNF39, LOC399959, PCDHGA1,* and *LGR6* are also significantly hypomethylated in CRC tissue compared to normal tissue in the TCGA dataset, suggesting that the changes observed in DNA methylation levels may be clinically important.

To our knowledge, our analysis is the first to assess the potential link between genome-wide DNA methylation in peripheral blood and future risk of CRC. Our analysis has revealed that there is potential in the use of peripheral blood-based DNA methylation profiling for CRC risk prediction. We have shown, with a ROC indicating good performance, an MRS model consisting of sixteen CpG panel that has the ability to differentiate CRC from healthy normal subjects.

One important strength of our study was its prospective design. The utilization of blood samples collected before diagnosis which indicated that the DNA methylation preceded the development of CRC by up to 6 years, enabled us to assess genome-wide measures of DNA methylation as potential biomarkers of risk as compared to measures of DNA methylation in retrospective designs which may have resulted from molecular changes due to carcinogenesis and medication.

A limitation of our study is its lack of replication. To the best of our knowledge, there are currently no other pre-diagnostic blood DNA Illumina Human Methylation 450 data for CRC studies available. However, we used TCGA dataset for external validation and recommend that other prospective cohort studies assess associations between genome-wide DNA methylation and CRC risk.

## Conclusion

Our study has identified a novel DNA methylation panel based on genome-wide analysis that is associated with CRC and suggests that differential peripheral blood DNA methylation panel may be an easily available biomarker for prediction of CRC risk in the future if validated in a prospective cohort. Further studies with larger cohort data will be needed to confirm this pattern.

## Supplementary information

**Additional file 1: Figure S1.** Volcano plot for differential DNA methylation analysis of all 399,934 CpG sites among 166 CRC and healthy normal subjects. The x-axis shows the mean DNA methylation difference (delta beta), while the y-axis shows the –log10 of the *p* value for each CpG.

**Additional file 2: Table S1.** Top Gene Ontology Enrichment Analysis of Differentially Methylated Genes of Nested Case Control Study Based on EPIC-Italy Cohort.

**Additional file 3: Table S2.** KEGG Pathway Enrichment Analysis of Nested Case Control Study Based on EPIC-Italy Cohort.

**Additional file 4: Table S3.** Differentially Methylated CpGs between Colorectal Cancer and Healthy Normal Subjects of Nested Case Control Study Based on EPIC-Italy Cohort.

**Additional file 5: Figure S2.** The distribution of the methylation values of the sixteen risk markers in CRC and healthy normal subjects in the training (a) and testing (b) dataset.

**Additional file 6: Table S4.** Univariate Analysis on the Associations of DNA Methylation Marker, MRS and Risk of CRC of Nested Case Control Study Based on EPIC-Italy Cohort.

**Additional file 7: Figure S3.** Box-plots of the MRS separately in CRC and healthy normal subjects for training dataset (Fig. S3a) and testing dataset (Fig. S3b).

**Additional file 8: Table S5.** Correlation between DNA Methylation Marker and Time-To-Diagnosis of Nested Case Control Study Based on EPIC-Italy Cohort.

**Additional file 9: Table S6.** Univariate Analysis on the Associations of DNA Methylation Marker, MRS and Risk of CRC TCGA dataset.

**Additional file 10: Figure S4.** Box-plots of the MRS separately in CRC and control subjects for TCGA dataset.

**Additional file 11: Figure S5.** Classification performance of methylation risk score (MRS) for CRC risk; Receiver operating characteristic (ROC) curve for methylation risk score (MRS) prediction of CRC, with area-under the curve (AUC) of 0.73 (95% CI: 0.66–0.79) on TCGA dataset.

## Data Availability

The raw data sets used to support the conclusions of this article are available on NCBI’s Gene Expression Omnibus (GEO) through GEO accession number GSE51032. https://www.ncbi.nlm.nih.gov/geo/query/acc.cgi?acc=GSE51032 and The Cancer Genome Atlas (TCGA, https://cancergenome.nih.gov/).

## Abbreviations

AUC: Area under the ROC curve
CI: Confidence interval
CRC: Colorectal cancer
DMPs: Differentially methylated probes
ESYT3: Extended Synaptotagmin 3
EPIC: European Prospective Investigation into Cancer and Nutrition
FDR: False discovery rate
GEO: Gene Expression Omnibus
GO: Gene ontology
KEGG: Kyoto Encyclopedia of Genes and Genomes
LASSO: Least absolute shrinkage and selection operator
LIMMA: Linear models for microarray data
LGR6: Leucine Rich Repeat Containing G Protein-Coupled Receptor 6
MRS: Methylation risk score
OR: Odds ratio
PCDHGA1: Protocadherin gamma subfamily A, 1
PPFIA3: PTPRF-interacting protein alpha 3
PTPN12: Protein tyrosine phosphatase non-receptor type 12
ROC: Receiver Operating Characteristic
