# Supplementary material for: A panel of DNA methylation signature from peripheral blood may predict colorectal cancer susceptibility
Source: BMC Cancer. 2020 Jul 25;20:692. doi: 10.1186/s12885-020-07194-5 (PMC7382833; doi:10.1186/s12885-020-07194-5)
Supplement: Supplementary file 1 — Additional file 1: Figure S1. Volcano plot for differential DNA methylation analysis of all 399,934 CpG sites among 166 CRC and healthy normal subjects. The x-axis shows the mean DNA methylation difference (delta beta), while the y-axis shows the –log10 of the p value for each CpG. [file 12885_2020_7194_MOESM1_ESM.docx]

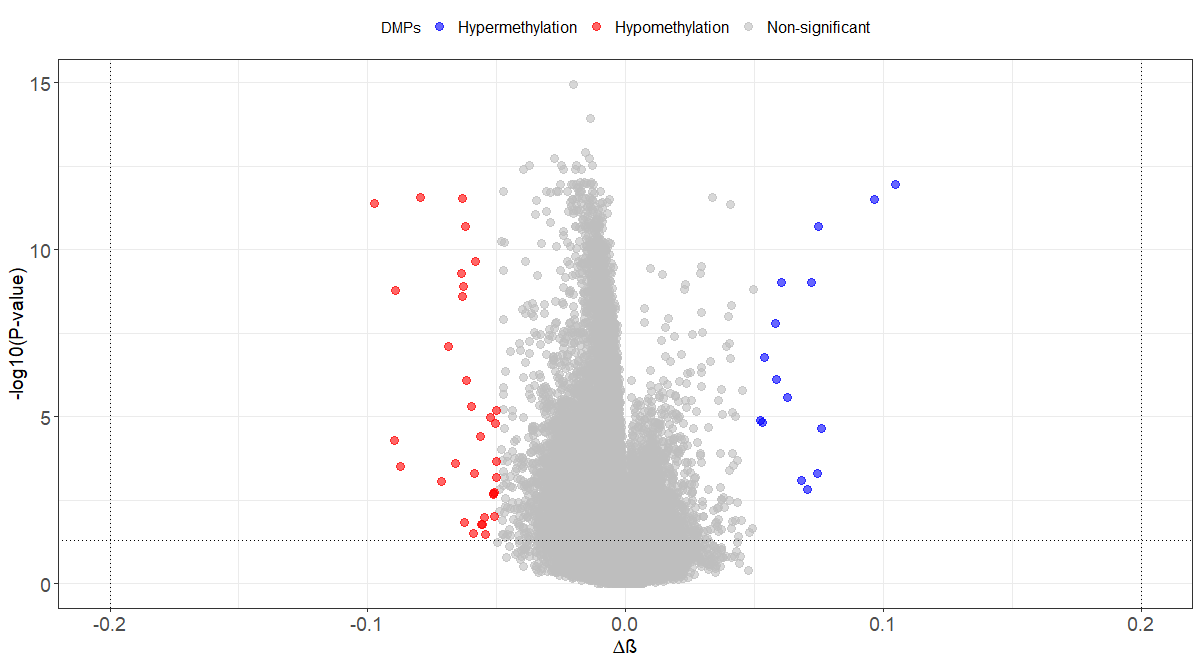


**Additional file 1: Figure S1.** Volcano plot for differential DNA methylation analysis of all 399,934 CpG sites among 166 CRC and healthy normal subjects. The x-axis shows the mean DNA methylation difference (delta beta), while the y-axis shows the –log10 of the p value for each CpG.
