# Supplementary material for: A panel of DNA methylation signature from peripheral blood may predict colorectal cancer susceptibility
Source: BMC Cancer. 2020 Jul 25;20:692. doi: 10.1186/s12885-020-07194-5 (PMC7382833; doi:10.1186/s12885-020-07194-5)
Supplement: Supplementary file 2 — Additional file 2: Table S1. Top Gene Ontology Enrichment Analysis of Differentially Methylated Genes of Nested Case Control Study Based on EPIC-Italy Cohort. [file 12885_2020_7194_MOESM2_ESM.docx]

**Table S1** Top Gene Ontology Enrichment Analysis of Differentially Methylated Genes of Nested Case Control Study Based on EPIC-Italy Cohort

| ID | Term | Ontology | Gene counts | *FDR* |
| --- | --- | --- | --- | --- |
| GO:0005515 | protein binding | MF | 11397 | 7.76E-57 |
| GO:0005737 | cytoplasm | CC | 11184 | 2.11E-45 |
| GO:0031234 | cytoplasmic part | CC | 9394 | 3.59E-42 |
| GO:0005488 | binding | MF | 14573 | 6.93E-42 |
| GO:0005622 | intracellular | CC | 14620 | 6.96E-41 |
| GO:0044424 | intracellular part | CC | 14311 | 3.78E-40 |
| GO:0071841 | cellular component organization or biogenesis | BP | 6285 | 1.40E-34 |
| GO:0043226 | organelle | CC | 13429 | 2.38E-34 |
| GO:0043229 | intracellular organelle | CC | 12623 | 3.89E-32 |
| GO:0016043 | cellular component organization | BP | 6114 | 7.02E-32 |
| GO:0098772 | molecular function | MF | 16827 | 4.92E-31 |
| GO:0044446 | intracellular organelle part | CC | 9351 | 1.14E-30 |
| GO:0044464 | cell part | CC | 16486 | 6.08E-30 |
| GO:0044422 | organelle part | CC | 9640 | 8.06E-30 |
| GO:0005623 | cell | CC | 16518 | 8.87E-30 |
| GO:0005829 | cytosol | CC | 4991 | 4.99E-29 |
| GO:0043227 | membrane-bounded organelle | CC | 12390 | 3.34E-28 |
| GO:0043231 | intracellular membrane-bounded organelle | CC | 10904 | 1.88E-25 |
| GO:0051641 | cellular localization | BP | 2798 | 7.77E-25 |
| GO:0005654 | nucleoplasm | CC | 3454 | 3.43E-24 |

Abbreviations: BP, Biological Process; CC, Cellular components; MF, Membrane function; FDR: false discovery rate
