# Supplementary material for: A panel of DNA methylation signature from peripheral blood may predict colorectal cancer susceptibility
Source: BMC Cancer. 2020 Jul 25;20:692. doi: 10.1186/s12885-020-07194-5 (PMC7382833; doi:10.1186/s12885-020-07194-5)
Supplement: Supplementary file 3 — Additional file 3: Table S2. KEGG Pathway Enrichment Analysis of Nested Case Control Study Based on EPIC-Italy Cohort. [file 12885_2020_7194_MOESM3_ESM.docx]

**Table S2** KEGG Pathway Enrichment Analysis of Nested Case Control Study Based on EPIC-Italy Cohort

| ID | Pathway | Gene counts | *FDR* |
| --- | --- | --- | --- |
| hsa04015 | Rap1 signaling pathway | 206 | 0.0004 |
| hsa04218 | Cellular senescence | 157 | 0.0012 |
| hsa01100 | Metabolic pathways | 1231 | 0.0012 |
| hsa04360 | Axon guidance | 180 | 0.0021 |
| hsa00970 | Aminoacyl-tRNA biosynthesis | 44 | 0.0032 |
| hsa04152 | AMPK signaling pathway | 119 | 0.0048 |
| hsa05200 | Pathways in cancer | 519 | 0.0066 |
| hsa04110 | Cell cycle | 124 | 0.0078 |
| hsa04115 | p53 signaling pathway | 72 | 0.0149 |
| hsa05203 | Human papillomavirus infection | 320 | 0.0161 |

Abbreviations: FDR, false discovery rate
