# Supplementary material for: A panel of DNA methylation signature from peripheral blood may predict colorectal cancer susceptibility
Source: BMC Cancer. 2020 Jul 25;20:692. doi: 10.1186/s12885-020-07194-5 (PMC7382833; doi:10.1186/s12885-020-07194-5)
Supplement: Supplementary file 4 — Additional file 4: Table S3. Differentially Methylated CpGs between Colorectal Cancer and Healthy Normal Subjects of Nested Case Control Study Based on EPIC-Italy Cohort. [file 12885_2020_7194_MOESM4_ESM.docx]

**Table S3** Differentially Methylated CpGs between Colorectal Cancer and Healthy Normal Subjects of Nested Case Control Study Based on EPIC-Italy Cohort

| CpG ID | Chr | Position | Gene Name | Gene Group | Relation to Island | Delta β^2^ | *Q- value*^3^ |
| --- | --- | --- | --- | --- | --- | --- | --- |
| cg00325531 | 1 | 75591353 | NA | NA | Island | -0.06 | 1.72E-02 |
| cg01066472 | 1 | 75591029 | NA | NA | Island | -0.05 | 9.78E-03 |
| cg06825163 | 1 | 202172912 | LGR6; LGR6; LGR6 | 1^st^ Exon; Body;  5'UTR | OpenSea | -0.05 | 1.00E-02 |
| cg12691488 | 1 | 243053673 | NA | NA | Island | 0.05 | 1.77E-07 |
| cg01620164 | 2 | 164590272 | FIGN | Body | N_Shelf | -0.07 | 8.59E-04 |
| cg16440561 | 2 | 220312854 | SPEG | Body | Island | -0.09 | 5.32E-05 |
| cg19015611 | 2 | 71126446 | VAX2 | TSS1500 | N_Shore | -0.06 | 8.40E-07 |
| cg20144008 | 2 | 173037423 | NA | NA | Island | -0.06 | 1.99E-11 |
| cg26309498 | 2 | 109607038 | EDAR | TSS1500 | OpenSea | -0.05 | 6.81E-06 |
| cg11643285 | 3 | 16411667 | RFTN1 | Body | OpenSea | -0.06 | 2.90E-12 |
| cg11885357 | 3 | 138152902 | ESYT3 | TSS1500 | N_Shore | -0.06 | 1.53E-02 |
| cg17238319 | 3 | 16428391 | RFTN1 | Body | OpenSea | -0.06 | 2.20E-10 |
| cg20432211 | 4 | 77342104 | NA | NA | Island | -0.05 | 2.14E-03 |
| cg12578536 | 5 | 43003251 | NA | NA | S_Shelf | -0.05 | 2.23E-04 |

Abbreviations: ^1^Chr, Chromosome; ^2^Delta β: Absolute mean β-value difference (delta beta ≥ 0.05); ^3^*Q- value*: False discovery rate, *(FDR* < 0.05)

**Table S3** Differentially Methylated CpGs between Colorectal Cancer and Healthy Normal Subjects of Nested Case Control Study Based on EPIC-Italy Cohort (Continued)

| CpG ID | Chr | Position | Gene Name | Gene Group | Relation to Island | Delta β^2^ | *Q- value*^3^ |
| --- | --- | --- | --- | --- | --- | --- | --- |
| cg18022036 | 5 | 19288941 | NA | NA | OpenSea | -0.07 | 8.14E-08 |
| cg05983326 | 5 | 140710688 | PCDHGA1;  PCDHGA1 | 1stExon; 1stExon | N_Shore | -0.05 | 2.00E-03 |
| cg01419670 | 5 | 115696151 | NA | NA | N_Shore | 0.06 | 9.82E-10 |
| cg00321614 | 5 | 172856932 | NA | NA | OpenSea | 0.06 | 7.57E-07 |
| cg10563109 | 6 | 5087749 | NA | NA | S_Shore | -0.05 | 1.08E-05 |
| cg15969227 | 6 | 26745279 | NA | NA | OpenSea | -0.06 | 5.09E-04 |
| cg16500605 | 6 | 31620861 | BAT3; BAT3;  BAT3; BAT3 | TSS1500;  TSS1500;  TSS1500;  TSS1500 | Island | -0.10 | 4.08E-12 |
| cg16170495 | 6 | 30042626 | RNF39;RNF39 | Body; Body | N_Shore | -0.05 | 1.62E-05 |
| cg16101278 | 6 | 6648823 | LY86 | Body | OpenSea | 0.10 | 1.14E-12 |
| cg06551493 | 7 | 77166702 | PTPN12; PTPN12; PTPN12 | TSS200; TSS1500;  TSS1500 | Island | -0.06 | 1.24E-09 |
| cg04205664 | 7 | 139209511 | CLEC2L | Body | S_Shore | 0.10 | 3.20E-12 |
| cg03151810 | 8 | 144371745 | NA | NA | Island | 0.07 | 1.48E-03 |
| cg03894796 | 8 | 144361315 | NA | NA | Island | 0.05 | 1.54E-05 |
| cg11240062 | 8 | 144361073 | NA | NA | Island | 0.08 | 2.26E-05 |
| cg11388673 | 8 | 144371779 | NA | NA | Island | 0.06 | 8.00E-04 |

Abbreviations: ^1^Chr, Chromosome; ^2^Delta β: Absolute mean β-value difference (delta beta ≥ 0.05); ^3^*Q- value*: False discovery rate, (*FDR* < 0.05)

**Table S3** Differentially Methylated CpGs between Colorectal Cancer and Healthy Normal Subjects of Nested Case Control Study Based on EPIC-Italy Cohort (Continued)

| CpG ID | Chr^1^ | Position | Gene Name | Gene Group | Relation to Island | Delta β^2^ | | *Q- value*^3^ |
| --- | --- | --- | --- | --- | --- | --- | --- | --- |
| cg09656541 | 10 | 115932823 | C10orf118 | 5'UTR | N_Shore | | 0.05 | 1.33E-05 |
| cg08829299 | 11 | 288305 | ATHL1 | TSS1500 | N_Shore | | -0.06 | 3.10E-02 |
| cg21585512 | 11 | 122030076 | LOC399959 | Body | OpenSea | | -0.07 | 2.46E-04 |
| cg21565415 | 11 | 618993 | MUPCDH;MUPCDH | Body;Body | S_Shore | | 0.07 | 9.91E-10 |
| cg24702253 | 11 | 3240068 | MRGPRG;C11orf36 | TSS200;Body | S_Shore | | 0.07 | 5.08E-04 |
| cg03691818 | 12 | 53085038 | KRT77 | Body | OpenSea | | -0.08 | 2.69E-12 |
| cg23256579 | 12 | 11002403 | PRR4;PRR4 | TSS1500;Body | OpenSea | | -0.09 | 1.62E-09 |
| cg16530981 | 12 | 42679156 | NA | NA | N_Shore | | 0.06 | 1.62E-08 |
| cg06710937 | 13 | 23489940 | NA | NA | Island | | -0.06 | 2.61E-09 |
| cg11057824 | 14 | 50471938 | C14orf182 | Body | S_Shore | | 0.06 | 2.65E-06 |
| cg12101586 | 15 | 75019203 | CYP1A1 | TSS1500 | Island | | -0.06 | 4.05E-05 |
| cg22549041 | 15 | 75019251 | CYP1A1 | TSS1500 | Island | | -0.05 | 6.73E-04 |
| cg26921482 | 16 | 2570283 | AMDHD2;AMDHD2 | TSS200;TSS200 | Island | | -0.06 | 5.26E-10 |
| cg04946709 | 16 | 59789030 | LOC644649 | Body | Island | | 0.07 | 1.99E-11 |
| cg17292758 | 19 | 49636594 | PPFIA3 | Body | Island | | -0.06 | 4.88E-06 |
| cg00157199 | 20 | 29551622 | NA | NA | Island | | -0.08 | 3.13E-04 |
| cg14815891 | 20 | 29611903 | FRG1B | Body | Island | | -0.06 | 1.67E-02 |
| cg17187762 | 22 | 28070120 | NA | NA | N_Shelf | | -0.05 | 1.83E-03 |

Abbreviations: ^1^Chr, Chromosome; ^2^Delta β: Absolute mean β-value difference (delta beta ≥ 0.05); ^3^*Q- value*: False discovery rate, (*FDR* < 0.05)
