# Supplementary material for: A panel of DNA methylation signature from peripheral blood may predict colorectal cancer susceptibility
Source: BMC Cancer. 2020 Jul 25;20:692. doi: 10.1186/s12885-020-07194-5 (PMC7382833; doi:10.1186/s12885-020-07194-5)
Supplement: Supplementary file 5 — Additional file 5: Figure S2. The distribution of the methylation values of the sixteen risk markers in CRC and healthy normal subjects in the training (a) and testing (b) dataset. [file 12885_2020_7194_MOESM5_ESM.docx]

**
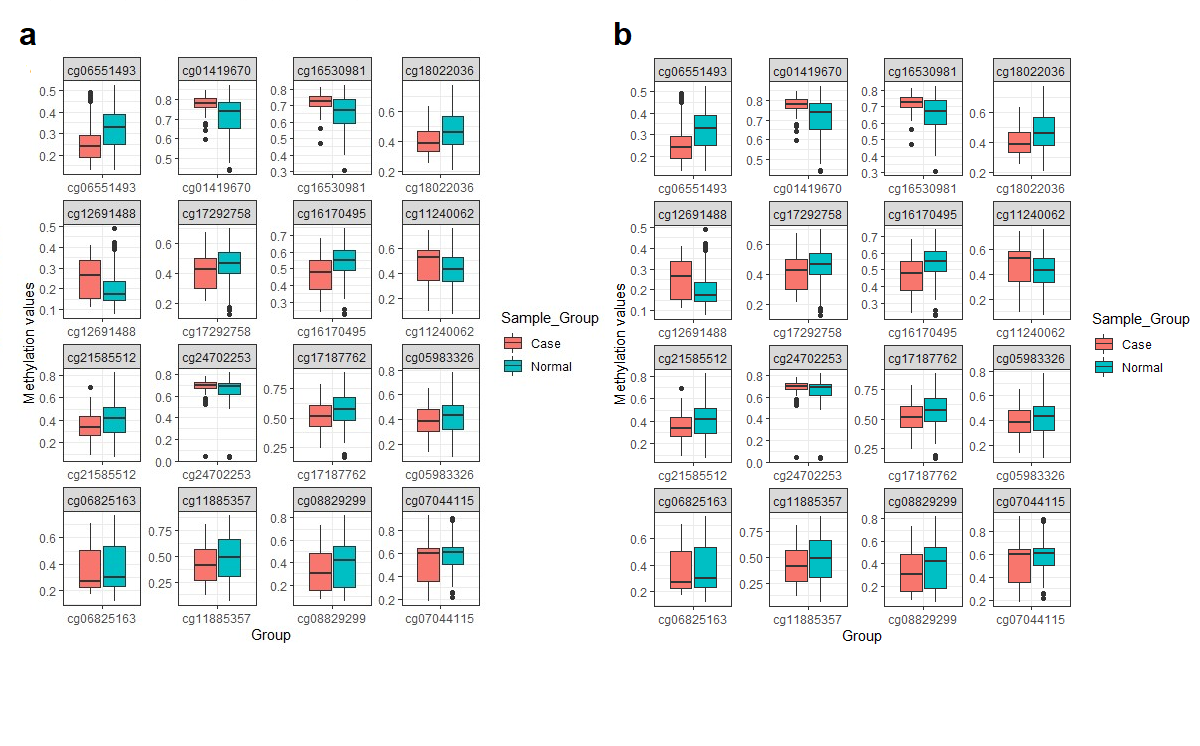
**

**Additional file 5:** **Figure S2.** The distribution of the methylation values of the sixteen risk markers in CRC and healthy normal subjects in the training (a) and testing (b) dataset.
