# Supplementary material for: A panel of DNA methylation signature from peripheral blood may predict colorectal cancer susceptibility
Source: BMC Cancer. 2020 Jul 25;20:692. doi: 10.1186/s12885-020-07194-5 (PMC7382833; doi:10.1186/s12885-020-07194-5)
Supplement: Supplementary file 6 — Additional file 6: Table S4. Univariate Analysis on the Associations of DNA Methylation Marker, MRS and Risk of CRC of Nested Case Control Study Based on EPIC-Italy Cohort. [file 12885_2020_7194_MOESM6_ESM.docx]

**Table S4** Univariate Analysis on the Associations of DNA Methylation Marker, MRS and Risk of CRC of Nested Case Control Study Based on EPIC-Italy Cohort

| CpG ID | Gene Name | Entire Dataset | | |  | Training Dataset | | |  | Testing Dataset | | |
| --- | --- | --- | --- | --- | --- | --- | --- | --- | --- | --- | --- | --- |
|  |  | OR | 95% CI | *P-value* |  | OR | 95% CI | *P-value* |  | OR | 95% CI | *P-value* |
| cg06551493 | PTPN12 | 0.49 | 0.39, 0.60 | **2.78e-11** |  | 0.58 | 0.45, 0.74 | **1.07e-05** |  | 0.30 | 0.19, 0.46 | **1.73e-07** |
| cg01419670 | NA | 2.51 | 1.94, 3.33 | **2.02e-11** |  | 2.64 | 1.94, 3.73 | **4.95e-09** |  | 2.22 | 1.43, 3.72 | **9.72e-04** |
| cg16530981 | NA | 2.23 | 1.76, 2.89 | **2.53e-10** |  | 2.36 | 1.77, 3.22 | **1.62e-08** |  | 1.95 | 1.28, 3.19 | **3.74e-03** |
| cg18022036 | NA | 0.52 | 0.42, 0.64 | **1.07e-09** |  | 0.52 | 0.40, 0.66 | **2.58e-07** |  | 0.53 | 0.36, 0.77 | **1.10e-03** |
| cg12691488 | NA | 1.74 | 1.46, 2.08 | **8.11e-10** |  | 1.52 | 1.23, 1.87 | **8.09e-05** |  | 2.43 | 1.73, 3.48 | **6.10e-07** |
| cg17292758 | PPFIA3 | 0.59 | 0.49, 0.72 | **6.68e-08** |  | 0.63 | 0.50, 0.79 | **4.82e-05** |  | 0.50 | 0.34, 0.72 | **2.94e-04** |
| cg16170495 | RNF39 | 0.62 | 0.51, 0.74 | **2.92e-07** |  | 0.65 | 0.52, 0.80 | **1.05e-04** |  | 0.55 | 0.38, 0.77 | **6.31e-04** |
| cg11240062 | NA | 1.71 | 1.41, 2.10 | **1.43e-07** |  | 1.64 | 1.30,2.09 | **3.76e-05** |  | 1.94 | 1.34 2.92 | **8.48e-04** |
| cg21585512 | LOC399959 | 0.64 | 0.52, 0.77 | **4.44e-06** |  | 0.54 | 0.42, 0.69 | **6.60e-07** |  | 0.92 | 0.66, 1.28 | 0.61 |
| cg24702253 | MRGPRG | 2.04 | 1.49, 3.02 | **6.94e-05** |  | 2.04 | 1.42, 3.26 | **6.77e-04** |  | 2.07 | 1.19, 4.79 | **3.82e-02** |
| cg17187762 | NA | 0.69 | 0.57, 0.83 | **7.23e-05** |  | 0.70 | 0.56, 0.87 | **1.46e-03** |  | 0.66 | 0.47, 0.92 | **1.67e-02** |
| cg05983326 | PCDHGA1 | 0.68 | 0.56, 0.82 | **8.39e-05** |  | 0.70 | 0.55, 0.87 | **1.64e-03** |  | 0.65 | 0.45, 0.92 | **1.75e-02** |
| cg06825163 | LGR6 | 0.72 | 0.59, 0.87 | **5.82e-04** |  | 0.70 | 0.56, 0.88 | **2.33e-03** |  | 0.75 | 0.53, 1.05 | 0.10 |
| cg11885357 | ESYT3 | 0.73 | 0.61, 0.88 | **9.91e-04** |  | 0.71 | 0.57, 0.88 | **2.14e-03** |  | 0.80 | 0.57, 1.12 | 0.20 |
| cg08829299 | ATHL1 | 0.75 | 0.62, 0.90 | **2.36e-03** |  | 0.76 | 0.61, 0.95 | **1.62e-02** |  | 0.72 | 0.50, 1.01 | 0.06 |
| cg07044115 | NA | 0.75 | 0.62, 0.90 | **1.86e-03** |  | 0.78 | 0.63, 0.97 | **2.61e-02** |  | 0.67 | 0.47, 0.93 | **0.02** |

Abbreviations: CI, confidence interval; CRC: colorectal cancer; MRS, methylation risk score
