# Supplementary material for: A panel of DNA methylation signature from peripheral blood may predict colorectal cancer susceptibility
Source: BMC Cancer. 2020 Jul 25;20:692. doi: 10.1186/s12885-020-07194-5 (PMC7382833; doi:10.1186/s12885-020-07194-5)
Supplement: Supplementary file 7 — Additional file 7: Figure S3. Box-plots of the MRS separately in CRC and healthy normal subjects for training dataset (Fig. S3a) and testing dataset (Fig. S3b). [file 12885_2020_7194_MOESM7_ESM.docx]

**
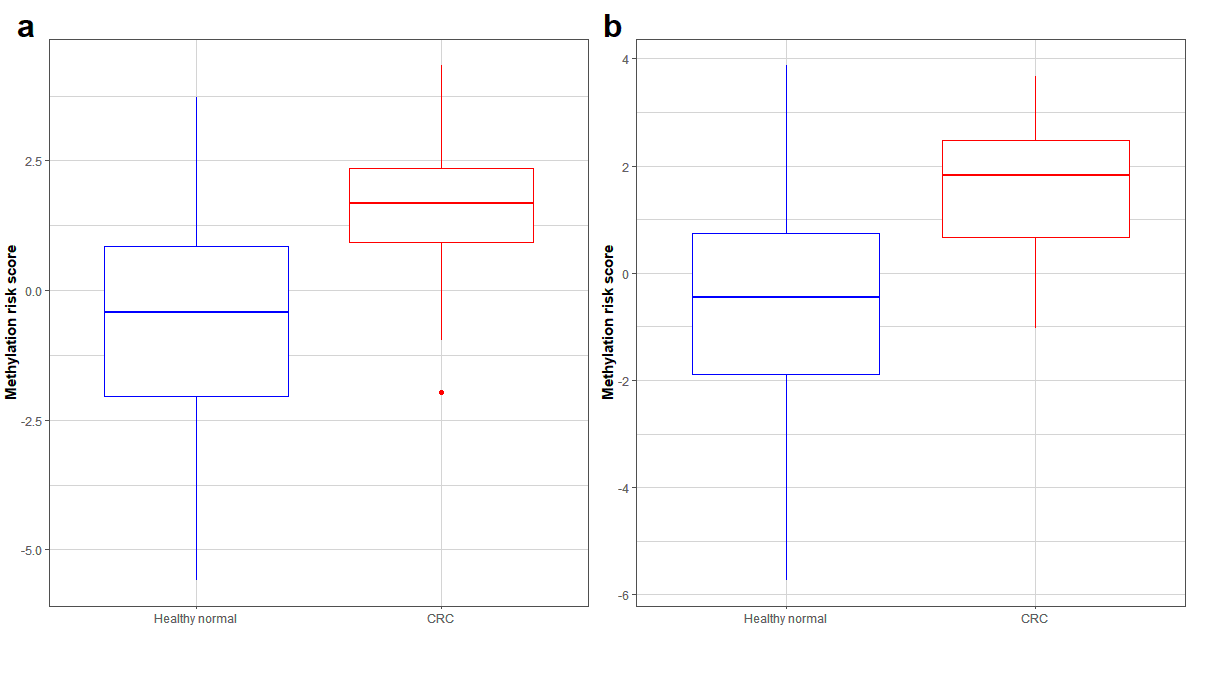
Additional file 7:** **Figure S3**. Box-plots of the MRS separately in CRC and healthy normal subjects for training dataset (Supplementary Figure. S3a) and testing dataset (Supplementary Figure. S3b).
