# Supplementary material for: A panel of DNA methylation signature from peripheral blood may predict colorectal cancer susceptibility
Source: BMC Cancer. 2020 Jul 25;20:692. doi: 10.1186/s12885-020-07194-5 (PMC7382833; doi:10.1186/s12885-020-07194-5)
Supplement: Supplementary file 8 — Additional file 8: Table S5. Correlation between DNA Methylation Marker and Time-To-Diagnosis of Nested Case Control Study Based on EPIC-Italy Cohort. [file 12885_2020_7194_MOESM8_ESM.docx]

**Table S5** Correlation between DNA Methylation Marker and Time-To-Diagnosis of Nested Case Control Study Based on EPIC-Italy Cohor**t**

| CpG ID | Gene Name | Estimate (r) | *P-value* |
| --- | --- | --- | --- |
| cg06551493 | PTPN12 | -0.10 | 0.18 |
| cg01419670 | NA | 0.12 | 0.11 |
| cg16530981 | NA | 0.08 | 1.07 |
| cg18022036 | NA | -0.16 | **0.03** |
| cg12691488 | NA | 0.15 | 0.06 |
| cg17292758 | PPFIA3 | -0.10 | 0.22 |
| cg16170495 | RNF39 | -0.02 | 0.79 |
| cg11240062 | NA | 0.17 | 0.03 |
| cg21585512 | LOC399959 | 0.03 | 0.67 |
| cg24702253 | MRGPRG | 0.06 | 0.43 |
| cg17187762 | NA | 0.01 | 0.92 |
| cg05983326 | PCDHGA1 | 0.11 | 0.15 |
| cg06825163 | LGR6 | -0.12 | 0.14 |
| cg11885357 | ESYT3 | -0.07 | 0.40 |
| cg08829299 | ATHL1 | -0.06 | 0.45 |
| cg07044115 | NA | 0.01 | 0.86 |
