# Supplementary material for: A panel of DNA methylation signature from peripheral blood may predict colorectal cancer susceptibility
Source: BMC Cancer. 2020 Jul 25;20:692. doi: 10.1186/s12885-020-07194-5 (PMC7382833; doi:10.1186/s12885-020-07194-5)
Supplement: Supplementary file 9 — Additional file 9: Table S6. Univariate Analysis on the Associations of DNA Methylation Marker, MRS and Risk of CRC TCGA dataset. [file 12885_2020_7194_MOESM9_ESM.docx]

**Table S6** Univariate Analysis on the Associations of DNA Methylation Marker, MRS and Risk of CRC The Cancer Genome Atlas

| CpG ID | Gene Name | Univariate | | |  | Multivariate | | |
| --- | --- | --- | --- | --- | --- | --- | --- | --- |
|  |  | OR | 95% CI | *P-value* |  | OR | 95% CI | *P-value* |
| cg06551493 | PTPN12 | 0.69 | 0.54, 0.90 | **0.004** |  | 0.67 | 0.52, 0.87 | **0.002** |
| cg01419670 | NA | 0.71 | 0.49, 1.02 | 0.07 |  | 0.97 | 0.49, 1.01 | 0.07 |
| cg12691488 | NA | 1.69 | 1.24, 2.30 | **0.0008** |  | 2.02 | 1.42, 2.94 | **0.0001** |
| cg17292758 | PPFIA3 | 3.49 | 2.59, 4.85 | **4.8e-15** |  | 3.84 | 2.79, 5.50 | **6.03e-15** |
| cg16170495 | RNF39 | 0.75 | 0.58, 0.98 | **0.02** |  | 0.74 | 0.58, 0.97 | **0.02** |
| cg11240062 | NA | 0.79 | 0.59, 1.07 | 0.12 |  | 0.75 | 0.55, 1.04 | 0.08 |
| cg21585512 | LOC399959 | 0.74 | 0.57, 0.98 | **0.03** |  | 0.69 | 0.52, 0.91 | **0.008** |
| cg24702253 | MRGPRG | 0.50 | 0.33, 0.73 | **0.0005** |  | 0.47 | 0.31, 0.69 | **0.0002** |
| cg17187762 | NA | 2.10 | 1.27, 3.95 | **0.01** |  | 2.00 | 1.23, 3.73 | **0.01** |
| cg05983326 | PCDHGA1 | 0.59 | 0.45, 0.77 | **0.0001** |  | 0.61 | 0.46, 0.81 | **0.0005** |
| cg06825163 | LGR6 | 0.93 | 0.66, 1.26 | 0.63 |  | 0.95 | 0.68, 1.28 | 0.72 |
| cg11885357 | ESYT3 | 2.21 | 1.72, 2.88 | **1.26e-09** |  | 2.61 | 1.97, 3.55 | **9.93e-11** |
| cg08829299 | ATHL1 | 1.18 | 0.84, 1.90 | 0.40 |  | 1.18 | 0.84, 1.88 | 0.41 |

Abbreviations: CI, confidence interval; CRC: colorectal cancer; MRS, methylation risk score; ORs adjusted for age and gender; *P values* < 0.05 are in bold
