# Supplementary material for: A panel of DNA methylation signature from peripheral blood may predict colorectal cancer susceptibility
Source: BMC Cancer. 2020 Jul 25;20:692. doi: 10.1186/s12885-020-07194-5 (PMC7382833; doi:10.1186/s12885-020-07194-5)
Supplement: Supplementary file 10 — Additional file 10: Figure S4. Box-plots of the MRS separately in CRC and control subjects for TCGA dataset. [file 12885_2020_7194_MOESM10_ESM.docx]

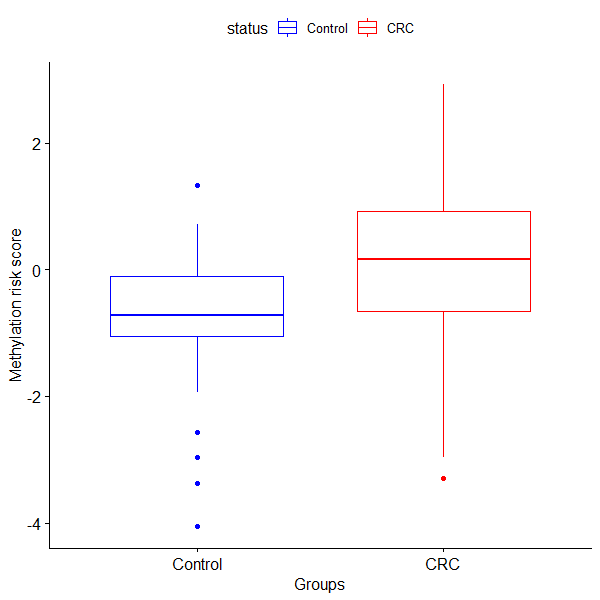
**Additional file 10:** **Figure S4**. Box-plots of the MRS separately in CRC and control subjects for TCGA dataset.
