# Supplementary material for: A panel of DNA methylation signature from peripheral blood may predict colorectal cancer susceptibility
Source: BMC Cancer. 2020 Jul 25;20:692. doi: 10.1186/s12885-020-07194-5 (PMC7382833; doi:10.1186/s12885-020-07194-5)
Supplement: Supplementary file 11 — Additional file 11: Figure S5. Classification performance of methylation risk score (MRS) for CRC risk; Receiver operating characteristic (ROC) curve for methylation risk score (MRS) prediction of CRC, with area-under the curve (AUC) of 0.73 (95% CI: 0.66–0.79) on TCGA dataset. [file 12885_2020_7194_MOESM11_ESM.docx]

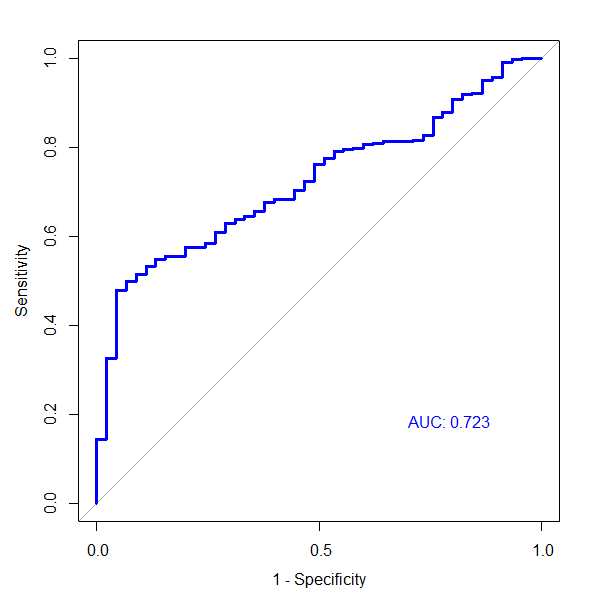
**Additional file 11:** **Figure S5**. Classification performance of methylation risk score (MRS) for CRC risk; Receiver operating characteristic (ROC) curve for methylation risk score (MRS) prediction of CRC, with area-under the curve (AUC) of 0.73 (95% CI: 0.66- 0.79) on TCGA dataset.
